# Practical Application of Toxicogenomics for Profiling Toxicant-Induced Biological Perturbations

**DOI:** 10.3390/ijms11093397

**Published:** 2010-09-20

**Authors:** Naoki Kiyosawa, Sunao Manabe, Takashi Yamoto, Atsushi Sanbuissho

**Affiliations:** 1 Medicinal Safety Research Laboratories, Daiichi Sankyo Co., Ltd., 717 Horikoshi, Fukuroi, Shizuoka 437-0065, Japan; E-Mails: yamoto.takashi.vw@daiichisankyo.co.jp (T.Y.); sanbuissho.atsushi.tv@daiichisankyo.co.jp (A.S.); 2 Global Project Management Department, Daiichi Sankyo Co., Ltd., 1-2-58, Hiromachi, Shinagawa, Tokyo 140-8710, Japan; E-Mail: manabe.sunao.u2@daiichisankyo.co.jp (S.M)

**Keywords:** toxicogenomics, microarray, biomarker, bioinformatics, systems biology

## Abstract

A systems-level understanding of molecular perturbations is crucial for evaluating chemical-induced toxicity risks appropriately, and for this purpose comprehensive gene expression analysis or toxicogenomics investigation is highly advantageous. The recent accumulation of toxicity-associated gene sets (toxicogenomic biomarkers), enrichment in public or commercial large-scale microarray database and availability of open-source software resources facilitate our utilization of the toxicogenomic data. However, toxicologists, who are usually not experts in computational sciences, tend to be overwhelmed by the gigantic amount of data. In this paper we present practical applications of toxicogenomics by utilizing biomarker gene sets and a simple scoring method by which overall gene set-level expression changes can be evaluated efficiently. Results from the gene set-level analysis are not only an easy interpretation of toxicological significance compared with individual gene-level profiling, but also are thought to be suitable for cross-platform or cross-institutional toxicogenomics data analysis. Enrichment in toxicogenomics databases, refinements of biomarker gene sets and scoring algorithms and the development of user-friendly integrative software will lead to better evaluation of toxicant-elicited biological perturbations.

## 1. Introduction

The term ‘toxic agent’ can be defined as any substance that causes harmful effects on living organisms, but in general, such hazardous effects are substantially dependent on the chemical’s exposure level. For instance, dietary salt may cause nephrotoxicity if an extreme amount is ingested at any time; however, do we consider sodium chloride a toxic agent? This is not the case because ordinarily we only need a spoon of salt for cooking and that amount will not harm healthy human bodies. In addition, in the case of pharmaceutical drugs, any chemical can be toxic when overdosed, and what makes them beneficial or toxic depends on their dose levels. Various types of preclinical toxicity studies are required before starting a human clinical trial to collect information on the toxicological profile such as target organs of toxicities, dose-response profile, recovery, toxicokinetics, genotoxicity, teratogenicity and carcinogenicity, using a sufficient number of experimental animals. However, even after a long and costly preclinical toxicity evaluation, species difference in mechanism of action (MOA) between humans and animals sometimes brings about unpredictable toxicities in humans [1]. Furthermore, a chemical once regarded as non-toxic could cause exaggerated toxicity in case undesirable chemical-chemical interaction occurs through inhibition or induction of ABC transporters [2] or hepatic drug metabolizing enzymes [3]. To evaluate and manage the potential hazardous risks of the chemicals, it is desirable to gain insight into the chemical-induced MOA in the target organ of toxicity, by which we can appropriately evaluate whether the chemical should be regarded as toxic or not according to its overall risk/benefit property.

For living organisms, it is sometimes necessary to modulate biological homeostasis to overcome potential hazardous effects caused by chemical exposure. For example, administration of anticonvulsant phenobarbital to rats causes liver hypertrophy, which is associated with induction of hepatic drug metabolizing enzymes such as CYP2B and CYP3A through the activation of the nuclear receptor CAR [4]. Although an increase in liver weight may look like a deleterious perturbation of hepatic homeostasis, toxicologists usually regard it as a non-toxic but rather a desirable response or adaptive response for the body. This is because such hepatic enzyme induction facilitates efficient metabolism and disposition of the exposed chemicals. In a systems-biological point of view, such liver system is called ‘robust’, not ‘homeostatic’ against phenobarbital exposure [5], where biological systems are not static but exhibit dynamic molecular reconstructions to maintain their cellular functional integrity. From this perspective, drug-induced toxicity can be defined as a ‘collapse of the biological robustness by drug exposure’. The recent advancement in Systems Biology is supported by dramatic advances in functional genomics techniques, especially the microarray technique by which expression levels of tens of thousands of genes can be measured simultaneously. Application of the microarray technique to toxicology research is called toxicogenomics (TGx), and is now widely utilized by pharmaceutical scientists in drug development [6]. The major problem in utilizing the TGx technique lies in its huge data size, as well as the complexity of the systems-level molecular interactions. We cannot avoid performing multivariate analyses to handle huge data sets, but toxicologists usually struggle to implement complicated statistical analysis. To overcome such difficulties, an easy, simple and practical analytical flow is desired. In this paper, we present practical methods of TGx data analysis for profiling chemical-elicited molecular perturbations using an open source analytical software, which will lead to better and easier utilization of TGx data to understand the MOA of toxicities.

## 2. Advancement of Toxicogenomics

### 2.1. Toxicogenomic Biomarker Gene Sets

**Figure 1:**
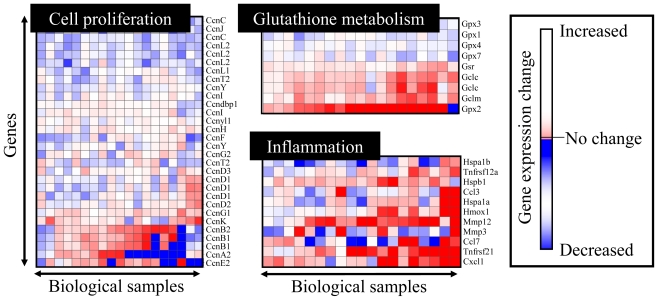
Expression profiling for toxicogenomic biomarker gene sets. Gene sets whose expression levels are closely associated with cell proliferation, glutathione metabolism and inflammatory responses are presented. The heat map represents gene expression changes, where up-regulation, no change and down-regulation are colored in red, white and blue, respectively.

In 2001, it was reported that the hepatic gene expression profiles in rats following treatment with various chemicals showed clear chemical-specific patterns when measured with the microarray technique [7,8]. Such chemical-specific changes in the transcriptome profile leads to changes in the proteome profile, the metabolome profile and eventually the tissue-level phenotypes. Thus, it is natural that the transcriptome profile would contain a significant degree of information for biological conditions at the moment, which may lead to a profound understanding of chemical-induced molecular perturbations. However, such chemical-specific gene expression data contain mixed molecular events that reflect complicated interactions among biological pathways such as xenobiotic metabolism, stress response, energy metabolism, protein synthesis/degradation, mRNA transcription/degradation, DNA repair/replication, cell proliferation/cell death control, *etc*. Microarray analysis measures tens of thousands of gene expression levels simultaneously, and is usually too complicated to appropriately interpret the significance of the gene expression changes at a time. Instead, it would be more practical to focus on the data for certain gene sets whose expression levels are closely associated with certain biological functions like glycolysis and cell proliferation. Such gene sets can be prepared from public information such as PubMed literature search, Gene Ontology [9], KEGG [10] and GenMAPP [11] biological pathway information. In addition, a number of gene sets have been reported whose expression levels are closely associated with certain toxicological endpoints, or TGx biomarker gene sets [12] such as cell injury [13], carcinogenicity [14,15], phospholipidosis [16,17] and glutathione depletion [18,19]. These TGx biomarkers can then be utilized for evaluation, diagnosis or prediction of toxicity based on their expression changes. As shown in Figure 1, it is much more informative and easy to interpret the microarray data by focusing on certain gene sets rather than observing whole data sets (*i.e.*, >30,000 gene probe sets in the case of Affymetrix GeneChip system). However, it is still too complicated when we need to handle a number of biomarker gene sets at a time. Because pharmaceutical toxicologists are usually not experts in handling vast amounts of data sets, it is crucial to develop user-friendly analytical software whose output is clear enough to interpret the toxicological significance.

### 2.2. Public and Commercial Microarray Database

A high quality and large-scale reference microarray database is desired for an appropriate interpretation of TGx data. A number of public databases are currently available, such as Gene Expression Ominibus (GEO) [20], ArrayExpress [21], Chemical Effects in Biological Systems (CEBS) [22], Comparative Toxicogenomics Database (CTD) [23] or EDGE [24]. In addition, the Toxicogenomics Project in Japan (http://wwwtgp.nibio.go.jp/index.html) and the InnoMed PredTox Consortium (http://www.innomed-predtox.com/) developed large-scale toxicogenomic databases, both of which contain microarray datasets for well-studied toxicants, as well as proprietary drugs using both *in vivo* and *in vitro* systems.

### 2.3. Open Source Software

Development of open source software is another driving factor of the recent advances in functional genomics research [25], although in many cases they require users to possess a certain degree of computational skills. In this paper, Bioconductor [26] (http://www.bioconductor.org/), which is implemented on the statistical software R [27] (http://cran.r-project.org/), GraphViz (http://www.graphviz.org/) and Cytoscape [28] (http://www.cytoscape.org/) were actually utilized and the results are presented.

## 3. Practical Application of TGx Database and Biomarkers

### 3.1. Scoring the Gene Set-Level Expression Changes

**Figure 2:**
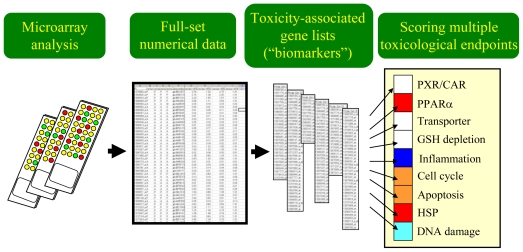
Scoring multiple toxicological endpoints using toxicogenomics data. Multiple toxicological endpoint-associated gene sets or TGx biomarkers need to be prepared in advance, and the overall expression changing levels for each gene set are calculated by certain algorithms such as the D-score, by which affected levels for each biological pathway can be evaluated intuitively.

As stated before, the process of data analysis and interpretation of the results becomes increasingly complex when we handle large-scale microarray data sets and multiple TGx biomarker gene sets simultaneously. To facilitate our understanding of the toxicological significance based on the TGx data, a simple scoring strategy was introduced to evaluate gene set-level expression changes. Figure 2 shows the general concept on how microarray data and multiple TGx biomarker gene sets can be processed to generate a simple score for each TGx biomarker gene set, by which toxicologists can identify which biological endpoints were affected by chemical exposure, and thereby can evaluate the toxicological significance efficiently.

In the Toxicogenomics Project in Japan, a large-scale TGx database called the Toxicogenomics Project-Genomics Assisted Toxicity Evaluation system (TG-GATEs) was developed that consisted of Affymetrix GeneChip data for approximately 150 prototypical toxicants on rat liver, kidney, hepatocytes and human hepatocytes [29]. In the TG-GATEs system, an expression ratio-based scoring method called ‘TGP1 score’ was utilized to facilitate understanding of the toxicological significance based on the TGx data set [30].

The TGP1 score is calculated by multiplying two elements: one element represents an index for the “overall direction of the expression change per probe set”, and the other represents the “overall magnitude of the expression change per gene” of the *Biomarker X* gene set. The sign of the first index will be either positive or negative when the overall expression changes of the genes in *Biomarker X* were up- or down-regulated, respectively, and will be expected to approach zero when the direction of expression changes is divergent. The second index is always positive and will be higher when the expression change levels of the genes show a higher value. Collectively, the TGP1 score will be higher when the genes included in *Biomarker X* show uniform up-regulation with higher expression changing levels.

### 3.2. Differentially-Regulated Gene Score (D-score)

The TGP1 score was found to be useful for efficient comprehension of large-scale microarray data sets. However, the TGP1 score calculation treats both low and high quality gene expression data equally, and therefore the calculated score would be flawed if low quality data was involved in the calculation. To overcome this shortcoming, we introduced a new scoring method called *Differentially-expressed gene score* (D-score) [31], where the data quality as well as the expression changing level for each gene is considered in the score calculation. Thus, the calculated score is much more reliable compared with the TGP1 score.

**Figure 3:**
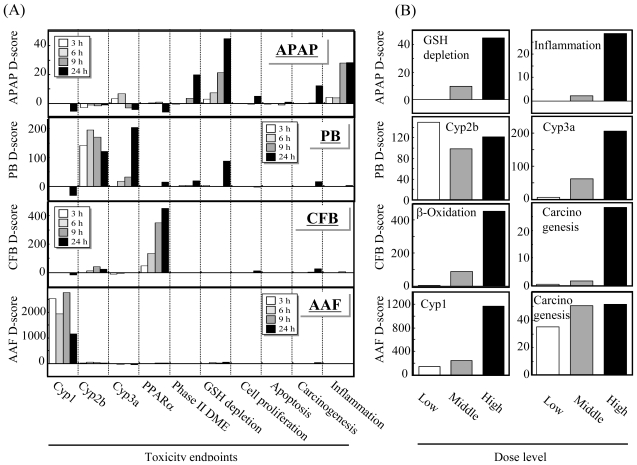
Detection of affected toxicological endpoints by D-score. (**A**) Rats were treated with prototypical hepatotoxicants acetaminophen (APAP), phenobarbital (PB), clofibrate (CFB) or acetamidofluorene (AAF), and the hepatic microarray data were obtained at 3, 6, 9 and 24 h after treatment. The D-score highlights the activated toxicological endpoints elicited by the chemicals: glutathione depletion and inflammation by APAP, Cyp2b/Cyp3a induction by PB, PPARα activation by CFB and Cyp1 induction by AAF; (**B**) All the D-scores except for that of Cyp2b exhibited a clear dose-response. Data are reprinted from [31] with permission from Elsevier.

Figure 3 represents D-scores for microarray data on rat livers treated with one of the four hepatotoxicants: acetaminophen (APAP), phenobarbital (PB), clofibrate (CFB) or acetamidofluorene (AAF) using 10 biomarker gene sets for score calculation. Stimulation of glutathione depletion and inflammation by APAP, induction of Cyp2b and Cyp3a genes, induction of PPARα by CFB and induction of Cyp1a genes are evident by D-score analysis results (Figure 3(A)), where the dose-dependent stimulation of these endpoints or genes can also be evaluated (Figure 3(B)). These results demonstrate that TGx biomarker gene sets and the D-score calculation method dramatically facilitate the interpretation of the TGx data.

### 3.3. Inference of Gene Set-Level Network Structure Using a TGx Database

Previous studies reported inconsistency of interlaboratory/inter-platform microarray results [32,33], while others have reported good concordance among laboratories [34–36] or inconclusive results [37,38]. Because a gene set-level or biological pathway-level analysis has been reported to be more robust and comparable for microarray data sets obtained from different studies [39,40], we hypothesized that the biological pathway-level interactions could be better evaluated using D-scores for multiple gene sets as compared with generic molecular network analysis methods, which were conducted with individual gene-by-gene-level analysis [41]. We inferred the gene set-level network structure using a large-scale TGx database, TG-GATEs, and a total of 58 gene sets [42] with a Gaussian graphical model (GGM) algorithm to calculate partial correlation coefficients among the gene sets [43]. In addition to gene expression data, we also included changing levels of phenotypic data such as organ weight, blood chemistry and hematology parameters for calculation.

**Figure 4:**
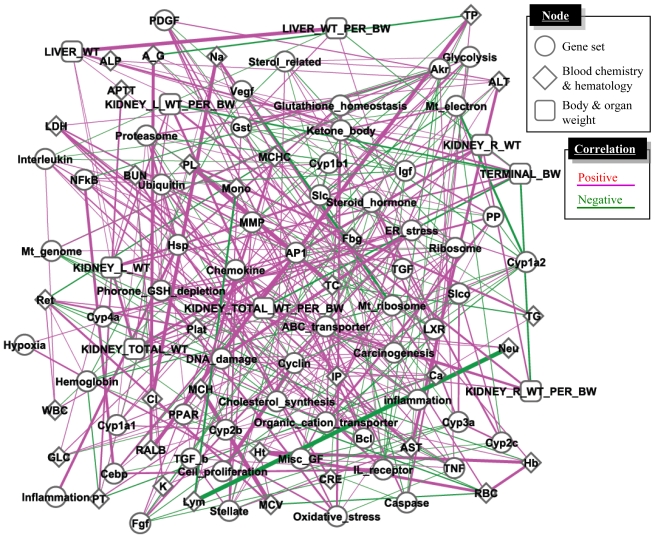
Gene set- and phenotype-level network analysis. A large-scale TGx database, TG-GATE, was used for extracting statistically significant relationships among gene sets and phenotypes by utilizing a GGM algorithm. The network consists of D-scores for 58 gene sets, as well as changing levels of phenotype data such as organ weight, blood chemistry and hematology. Purple and green represent positive and negative partial correlation coefficients, respectively, and the width of the lines represents strength of the correlation measured with partial correlation coefficient. The network was drawn with open-source software Cytoscape.

The inferred network presented in Figure 4 was found to contain a number of toxicologically-relevant gene set—gene set and gene set-phenotype relationships, such as blood glucose level and hepatic glycolysis-associated gene sets, or blood aminotransferase enzyme activity and inflammation-associated gene sets [42]. These results demonstrate that the retrospective network inference using a GGM algorithm successfully highlighted toxicologically significant gene set- and phenotype-level relationships from a large-scale TGx database. Furthermore, microarray data set obtained outside TG-GATEs was found to be well compatible with the network structure inferred based on TG-GATEs [42], suggesting that the gene set-level network structure was robust enough to be applicable for external microarray data sets.

## 4. Case Study: Bromobenzene-Induced Molecular Perturbation

**Figure 5:**
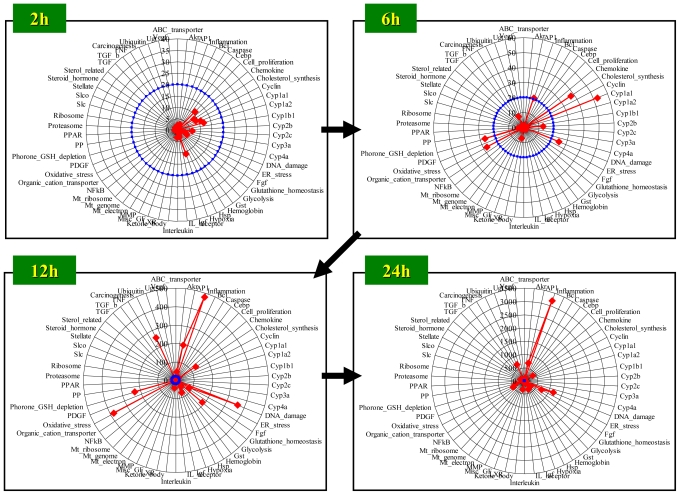
Time course of D-score: Radar chart presentation. D-scores were calculated for rat livers harvested at 2, 6, 12 and 24 h after bromobenzene treatment, and are presented in a radar chart. The red line indicates the D-score for each gene set, and the blue circle indicates a D-score = 20 for each gene set. Detailed information for the gene sets used can be obtained in a previous report [42].

**Figure 6:**
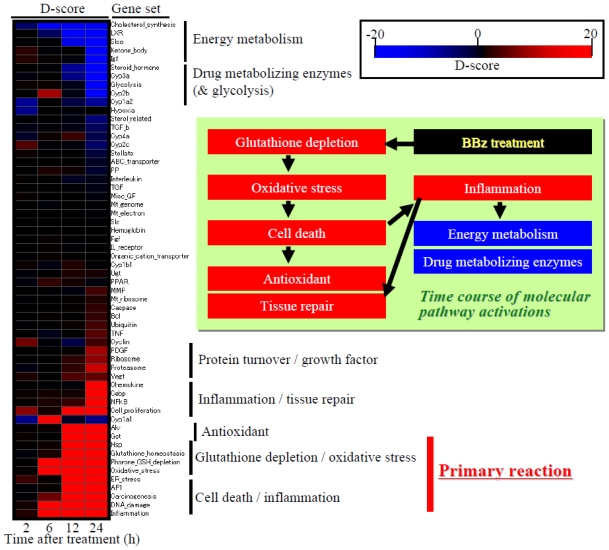
Time course of D-score: Heat map presentation. Red and blue indicate high and low D-scores, or up- and down-regulation for each gene set, respectively. The heat map indicates that the first trigger invoked by bromobenzene exposure was glutathione depletion and associated oxidative stress responses, followed by cell death, inflammation and up-regulation of antioxidant factors, as well as down-regulation of energy metabolism and drug metabolizing enzymes.

**Figure 7:**
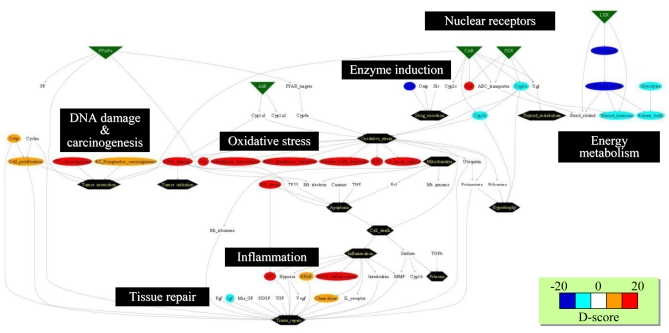
Network structure presentation of D-scores. Biological and toxicological relationships among gene sets were visualized as a supervised network using GraphViz software. The D-scores calculated for microarray data on rat livers at 24 h after bromobenzene treatment are presented in a network structure using GraphViz software, where red and blue indicate high and low D-scores, respectively. The network demonstrates oxidative stress and DNA damage were induced by bromobenzene treatment, while sterol metabolism was down-regulated.

In this section, a case study is presented for evaluating hepatic molecular perturbations elicited by 300 mg/kg bromobenzene (BBz) treatment in rats. D-score was calculated for a total of 58 gene sets and the calculated score was presented as either a radar chart (Figure 5), heat map (Figure 6) or network structure (Figure 7).

### 4.1. Radar Chart Presentation

Figure 5 shows the time course of absolute D-score values presented in radar charts, where a total of 58 gene sets, a detailed gene list which has been reported previously [42], were used for score calculation. Significant biological perturbations became evident at 6 h after BBz treatment, where *DNA damage*- and *Glutathione depletion*-associated gene sets exhibited high D-scores. BBz is reported to cause hepatic glutathione depletion through the generation of reactive metabolites [44], which is concordant with the high D-score for the glutathione depletion-associated genes. At 12 h, D-scores for *Oxidative stress*- and *Inflammation*-associated gene sets exhibited high scores, suggesting that oxidative stress, supposedly associated with glutathione depletion, induced liver injury, which was followed by an inflammatory response. *Gluthatione homeostasis*-associated genes, which include glutathione synthesis-related genes, were also activated at 12 h, which would contribute to feedback up-regulation of glutathione synthesis against acute glutathione depletion. The D-scores for *Inflammation*- and *DNA damage*-associated gene sets became considerably high at 24 h after BBz treatment, suggesting the level of liver injury progressed in spite of activation of *Glutathione homeostasis*-associated genes.

### 4.2. Heat Map Presentation

Time course transition of D-score profiles is presented in Figure 6, which clearly demonstrates the molecular mechanisms of BBz-elicited toxicity: Glutathione depletion and oxidative stress are the first triggers after BBz treatment, which caused cell death suggested by up-regulation of DNA damage-associated genes. This was followed by inflammation, tissue repair, as well as activation of antioxidant factors such as up-regulation of glutathione synthesis-related genes, glutathione *S*-transferase (Gst) genes or aldo-keto reductase (Akr) genes.

### 4.3. Supervised Network Structure Presentation

The BBz-elicited biological responses can also be visualized with the pre-defined biological network structure. Figure 7 represents a D-score profile at 24 h after BBz treatment, where gene sets associated with glutathione depletion, oxidative stress and inflammation were up-regulated (colored in red). On the other hand, gene sets associated with energy metabolism (*i.e.*, cholesterol synthesis and glycolysis) were down-regulated (colored in blue). Thus, the systems-level biological responses can be intuitively characterized with the supervised gene set-level biological network. However, the network structure should be kept up-to-date in a timely manner when novel toxicological knowledge has been obtained.

## 5. Conclusion

**Figure 8:**
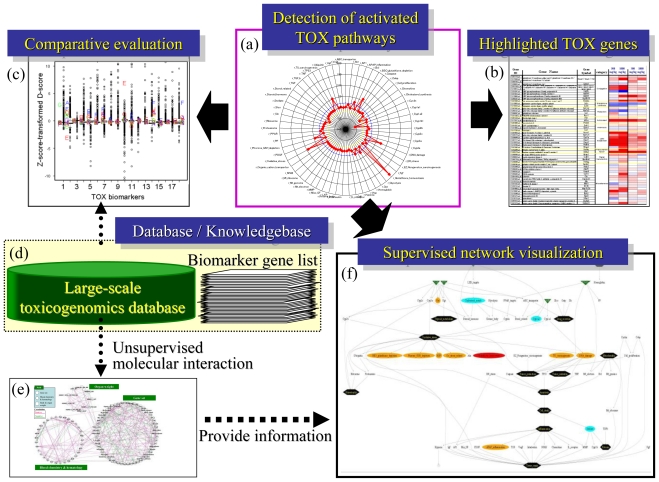
Analytical flow for toxicity evaluation using TGx data. (**A**) Radar chart for D-scores using 58 gene sets; (**B**) Box plot for comparative analysis using a reference TGx database; (**C**) Heat map for individual genes; (**D**) TGx reference database and TGx biomarker knowledgebase; (**E**) Unsupervised gene set-level network inference to extract toxicological relationships among pathways; (**F**) Supervised gene set-level network.

Systems-level understanding of molecular perturbations is crucial for evaluating chemical-induced toxicity risks. Microarray data provides comprehensive gene expression responses against chemical exposure, and therefore the TGx approach is highly advantageous for understanding systems-level biological perturbations. To solve the difficulty in handling huge amounts of TGx data sets, preparation of TGx biomarker gene sets and implementation of gene set-level data analysis are effective. The general flow of TGx data analysis is shown in Figure 8. The first step is to identify biological pathways that were affected by the chemical exposure, for which a radar chart presentation will be useful (Figure 8(A)). Detailed expression analysis of the individual genes will be needed to focus on the affected biological pathways (Figure 8(B)). When available, large-scale TGx reference database will be utilized for comparative analysis to appropriately evaluate the toxicological significance (Figures 8(C) and 8(D)). To comprehend the systems-level molecular dynamics, relationships among pathways should be taken into consideration (Figure 8(F)). Such analytical flow can be automated if appropriate computational skills are available. Refining toxicogenomic biomarker gene sets, scoring algorithm and development of user-friendly integrative software will substantially help the utilization of the TGx data set to evaluate biological response by which hazardous effects of exposed chemicals could be appropriately managed.
